# A tradeoff between physical encounters and consumption determines an optimal droplet size for microbial degradation of dispersed oil

**DOI:** 10.1038/s41598-022-08581-7

**Published:** 2022-03-18

**Authors:** Vicente I. Fernandez, Roman Stocker, Gabriel Juarez

**Affiliations:** 1 Department of Civil, Environmental, and Geomatic Engineering, Institute of Environmental Engineering, Eidgenossische Technische Hochschule (ETH) Zurich, 8093 Zurich, Switzerland; 2 Mechanical Science and Engineering Department, University of Illinois at Urbana-Champaign, Urbana, IL 61801 USA

**Keywords:** Environmental impact, Natural hazards, Statistical physics, thermodynamics and nonlinear dynamics, Biological physics

## Abstract

Immiscible hydrocarbons occur in the ocean water column as droplets of varying diameters. Although microbial oil degradation is a central process in the remediation of hydrocarbon pollution in marine environments, the relationship between droplet size distribution and oil degradation rates by bacteria remains unclear, with a conflicting history of laboratory studies. Despite this knowledge gap, the use of chemical dispersants in oil spill response and mitigation is based on the rationale that increasing the surface-area-to-volume ratio of droplets will enhance net bacterial biodegradation rates. We demonstrate that this intuitive argument does not apply to most natural marine environments, where the abundance of oil droplets is much lower than in laboratory experiments and droplet-bacteria encounters are the limiting factor. We present a mechanistic encounter-consumption model to predict the characteristic time for oil degradation by marine bacteria as a function of the initial oil concentration, the distribution of droplet sizes, and the initial abundance of oil-degrading bacteria. We find that the tradeoff between the encounter time and the consumption time leads to an optimal droplet size larger than the average size generated by the application of dispersants. Reducing droplet size below this optimum can increase the persistence of oil droplets in the environment from weeks to years. The new perspective granted by this biophysical model of biodegradation that explicitly accounts for oil–microbe encounters changes our understanding of biodegradation particularly in the deep ocean, where droplets are often small and oil concentrations low, and explains degradation rate discrepancies between laboratory and field studies.

## Introduction

When petroleum hydrocarbons are released into marine environments, whether from natural or anthropogenic sources, they persist as oil droplets in the water column for long periods of time. Exactly how long and what the typical fate of a droplet is remain open questions, but bacterial degradation is acknowledged as one of the primary mechanisms by which oil droplets are removed from the environment, with some marine bacteria specializing in hydrocarbon degradation^1,2^. For example, bacteria were estimated by one study to be responsible for 43–61% of hydrocarbon degradation in the Deepwater Horizon spill^3^. The role of bacteria in degrading oil droplets has deeply influenced the response to oil spills, promoting the use of chemical dispersants on surface oil slicks^4,5^ and at depth^6^ to reduce droplet sizes, based on the rationale that the increase in the surface-area-to-volume ratio of droplets favors their degradation by bacteria^7^.

A mechanistic understanding of the process by which bacteria degrade oil in the ocean is hampered by its biological, chemical, and environmental complexity. There is a well-developed literature on models of degradation that account for transport at the scale of the water column^8–10^ for the role of different bacteria^11–13^ for the range of different hydrocarbon components associated with oil spills^11,14,15^, and for other physical processes, especially photo-oxidation^13,15,16^. While the available surface area of the droplet-phase oil features as an explicit parameter in some models^16,17^, no model to date accounts for the biophysics at the scale of individual droplets and bacteria, in favor of a focus on the large, environmental scale of oil degradation. Direct measurements in the field are difficult because the concentration of oil droplets is typically very low, driven by natural mixing processes^18,19^, and droplets are exceedingly difficult to track individually. Laboratory studies are subject to a different set of challenges, in reproducing natural environmental conditions, maintaining experiments long enough to observe significant degradation^18,20^, and utilizing sufficient oil for chemical analysis^18^. As a result, laboratory measurements yield a very broad range of degradation rates and are highly sensitive to experimental conditions^18^.

A key biophysical component of oil degradation that has been overlooked to date is the initial process by which bacteria encounter and attach to oil droplets. In studies that consider the role of the oil surface area in the biodegradation process, the entire surface area of the droplet dispersion is assumed to be immediately available for bacterial growth, i.e., bacteria-oil encounters are assumed to be instantaneous^16,17^. However, even in highly oil enriched environments such as the middle of the underwater plume that developed from the tremendous amount of subsea dispersant injection during the Deepwater Horizon accident^21^, the concentration of oil only reached a maximum of 10 parts per million (ppm)^22^, corresponding to approximately 10^6^ droplets per liter with a mean diameter of 20 µm^23,24^ and thus, to an average distance between oil droplets of 1 mm. In these circumstances, it would take non-motile bacteria 280 d to encounter a droplet based on random Brownian motion and even highly motile bacteria 21 h (Supplementary information). Here we present the results of a new, physically grounded mathematical model termed the microscale oil degradation model (MODEM) that focuses on the biophysical interactions and biodegradation to show that encounters are often a major limiting factor for biological oil degradation in natural environments. Our findings indicate that efforts to enhance biodegradation by decreasing droplet sizes, for example by means of dispersants, can be highly counterproductive, particularly at depth.

## Results

### The microscale oil degradation model (MODEM)

**Figure 1:**
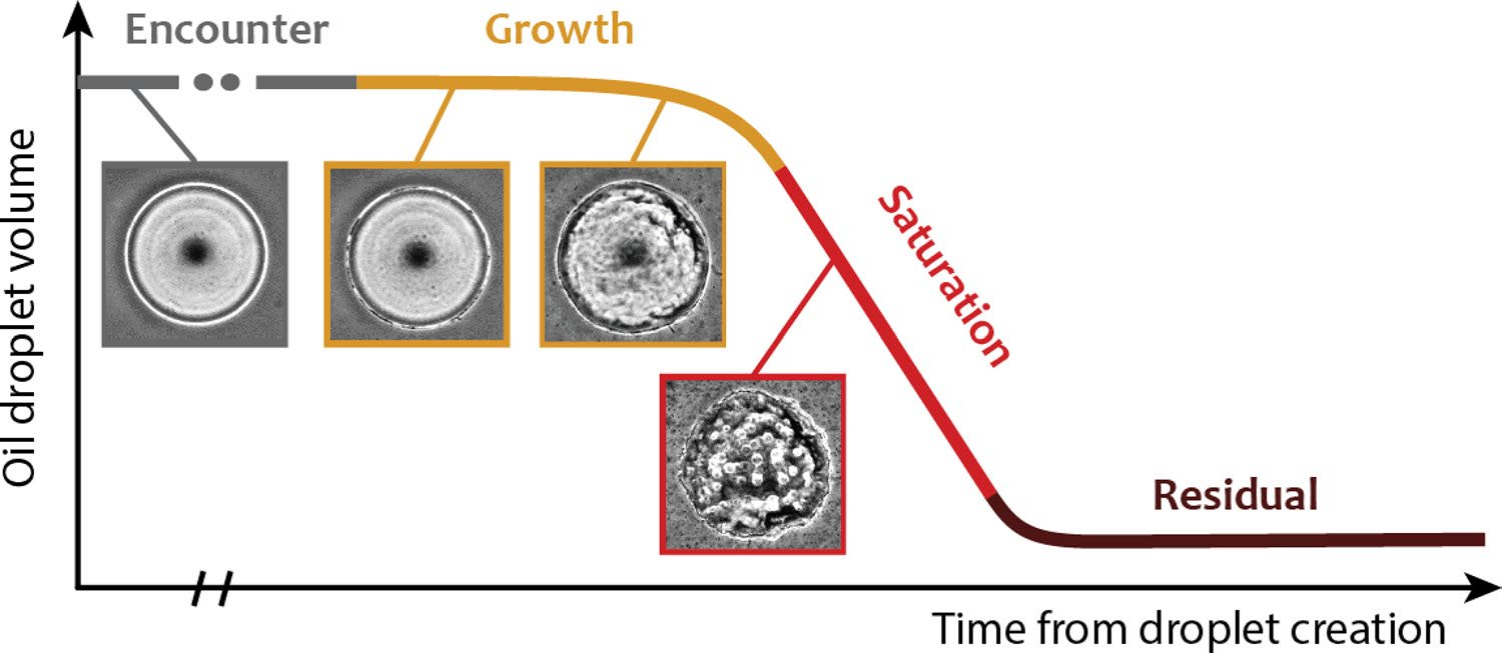
Schematic of the life of an oil droplet. The microbial degradation of an oil droplet is divided into four stages (time not to scale). During the initial encounter stage, the droplet does not have attached oil-degrading bacteria. After the first bacterial attachment, bacterial growth occurs until the available surface area is covered, inducing a transition to the saturation stage and linear degradation. In the final, residual stage, a non-zero volume can remain (non-metabolizable components). The insets are illustrative images of the stages taken from experiments using phase contrast time-lapse microscopy of an oil-degrading bacteria encountering and consuming a 100 µm diameter crude oil droplet.

We divide the biodegradation process of an oil droplet (radius *R*, mass *M*_*o*_), after it enters the water column, into four stages (Fig. 1). These four stages were motivated by observations in experiments where time-lapse microscopy was used to directly monitor the encounters and growth of oil-degrading bacteria at the midplane of a 100 µm diameter crude oil droplet^25^, and are consistent with previous observations^26–33^. The inset images are for illustrative purposes to motivate the modeling stages (details in Supplementary information). In the *encounter* stage, we consider the droplet—assumed neutrally buoyant and initially free of oil-degrading bacteria—and a single species of non-motile, oil-degrading bacteria, suspended in a quiescent fluid environment. For simplicity, we assume that the droplet-phase oil is composed of two components: one metabolizable by the modeled bacteria and one non-metabolizable^34^. During this first stage, the droplet radius and mass do not change, as there is no biodegradation. The *growth* stage begins when the first oil-degrading bacterium encounters and attaches to the oil droplet surface, beginning to transform hydrocarbons into biomass. As bacteria divide, they are retained at the droplet surface because of interfacial tension, leading to exponential growth of the colony with time^25,35^, and hence exponential degradation of the metabolizable fraction of the oil droplet (Fig. 1). The third stage, the *saturation* stage, begins once bacteria have reached the maximum capacity of the oil droplet surface. We assume that once the droplet surface is saturated with bacteria, additional bacteria generated from the biodegradation of the oil return to a planktonic state and can encounter other droplets^28,36,37^. As a result, the saturation stage is characterized by a constant rate of oil biodegradation (Supplementary information). We also refer to the growth and saturation stages taken together as the *consumption* phase. In the final stage, the *residual* stage, biodegradation of the oil droplet ceases, leaving a (non-metabolizable) fraction of the original oil mass and the colonizing cells in a small pellet^34^. We implement these four stages in a new, microscale oil degradation model (MODEM), which we use to predict large-scale oil degradation dynamics under different environmental conditions. We first consider the average oil droplet, then account for the distribution of droplet sizes.

### The average oil droplet

For an isolated oil droplet, MODEM yields an explicit formula for the average microbial biodegradation time of the droplet,1obtained as the sum of the timescales associated with each stage in the biodegradation process. The first term (‘Encounter’) describes the average time for the first encounter between diffusing bacteria and the oil droplet^38^, where *d*_*D*_ is the initial diameter of the spherical oil droplet and *d*_B_ is the equivalent diameter of a bacterium. The diffusivities, *D*_*D*_ and *D*_*B*_, are computed using the Stokes–Einstein relation for the oil droplet and bacteria, respectively, and *C*_*B*_ is the concentration of oil-degrading bacteria in the water (assumed constant for the time being). The denominator of the encounter term is the encounter rate (λ). The second term (‘Growth’) corresponds to the duration of exponential growth of bacteria on the droplet surface, with doubling time τ. The growth stage is limited by the maximum number of bacteria, *B*_*max*_, that can colonize the oil droplet surface, determined either by the saturation of the surface area (based on the initial droplet size) or by the complete biodegradation of the droplet, whichever occurs first. For droplets large enough that their surface is covered in bacteria before the droplet is degraded, *B*_*max*_ depends on the maximum surface area coverage fraction, which is measured relative to the initial droplet size *d*_*D*_ (hence *B*_*max*_ is proportional to *d*_*D*_^2^; Supplementary Table 1). The maximum surface area coverage fraction can exceed 1 in case of deformations of the oil–water interface or of attachment of more than a monolayer of bacteria to the surface (e.g., biofilm formation). During the saturation stage, we assume that the number of bacteria on the droplet remains *B*_*max*_ for the duration of the biodegradation and that all further progeny is released into the water column. The final term (‘Saturation’) quantifies the time for the remaining metabolizable oil to be degraded by the *B*_*max*_ bacteria (hence degradation rate is constant in this stage). *M*_*o*_, proportional to *d*_*D*_^3^, is the initial metabolizable oil mass expressed in units of equivalent bacteria (Supplementary Table 1).

**Figure 2:**
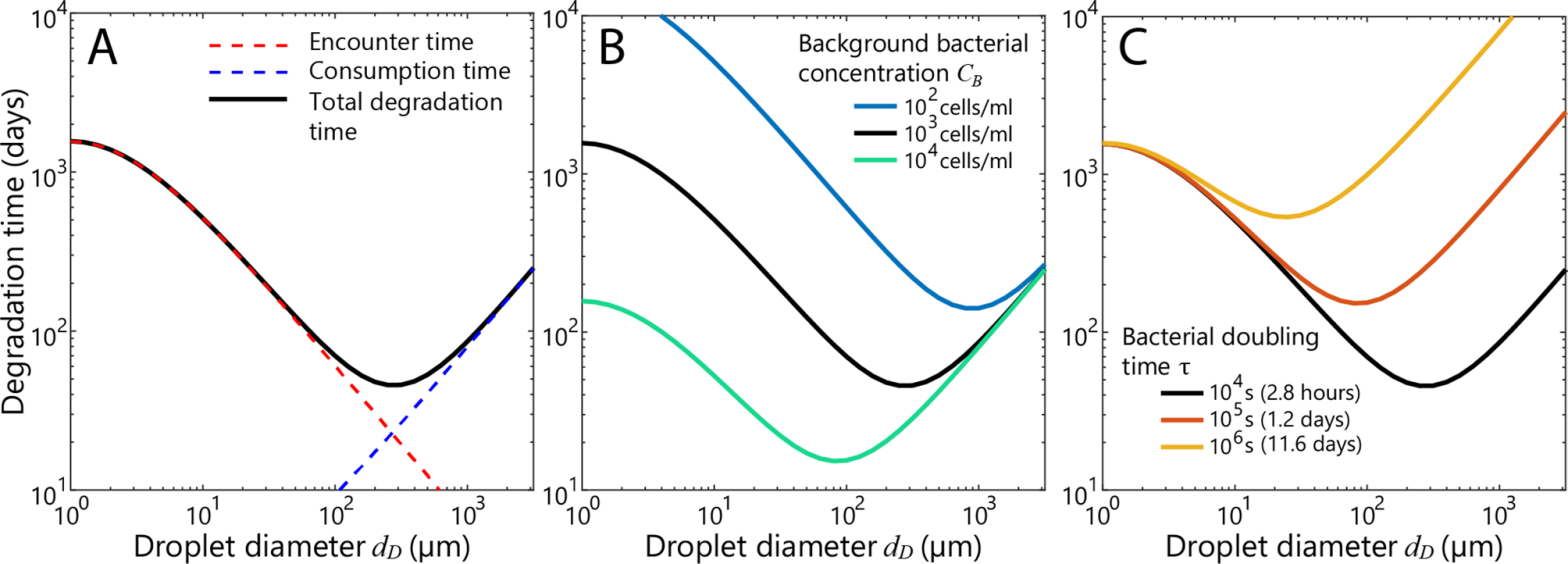
Biodegradation time of an average single oil droplet. **(a)** The average biodegradation time for a single droplet described by Eq. (1), using the environmental and biological parameters from Supplementary Table 1. The contributions from the encounter time (red dashed line) and the consumption time (blue dashed line) illustrate the tradeoff of the two processes in terms of droplet diameter. **(b)** Environmental parameters, here the background bacterial concentration *C*_*B*_, can have a major effect on the encounter-driven contribution to the biodegradation time (the portion of the curves left of the minimum). **(c)** Biological parameters, here the bacterial doubling time, can have a major effect on the consumption-driven contribution to the biodegradation time (the portion of the curves right of the minimum). The environmental and biological parameters of the system alter the droplet size for which the biodegradation time is smallest.

The average microbial degradation time for deep-water droplet sizes (10 µm to 1 mm^39–41^) is governed by a tradeoff between two processes: encounters and consumption (Fig. 2). The total biodegradation time for an average oil droplet in a population of equally sized droplets is simply the sum of the encounter time and the consumption time (Eq. 1). The average encounter time increases with decreasing droplet size (Fig. 2a, red dashed line). In contrast, the average single-droplet consumption time decreases with decreasing droplet size (Fig. 2a, blue dashed line), due to the increased surface-area-to-volume ratio. The latter well-known relationship is often used to justify the use of dispersants as accelerating biodegradation^7^. However, depending on the size of the oil droplets, the total biodegradation time may be primarily driven by encounters (encounter-limited), not by consumption (consumption-limited).

The tradeoff between droplet encounters and consumption leads to a pronounced local minimum in the average biodegradation time (Eq. 1) representing the optimal droplet size for biodegradation (Fig. 2a). Based on model parameters representative of the deep ocean and a doubling time of bacteria on oil of τ = 2.8 h (Supplementary Table 1), the average-droplet analysis predicts the optimal droplet diameter to be 275 µm, for which the average biodegradation time is 46 days. Reducing and increasing droplet size away from the optimal size both lead to a nearly proportional increase in the average biodegradation time. For example, the average biodegradation time for a 27 µm droplet is 217 days and for a 2750 µm droplet it is 218 days (Fig. 2a).

The optimal average droplet diameter depends on the concentration of oil-degrading bacteria in the water column and their growth rate on the oil (Fig. 2b,c). The concentration of bacteria in the water column, *C*_*B*_, has a strong influence on the biodegradation time by affecting the encounter rate (Eq. 1). For a low background concentration of 10^2^ cells/ml (with the remaining parameters as in Supplementary Table 1) the optimal droplet size is nearly 1 mm, whereas for a high concentration of 10^4^ cells/ml it is approximately 100 µm (Fig. 2b). In contrast, the doubling time of bacteria on oil, τ, affects the biodegradation time in the consumption stage (Eq. 1). When the doubling time on oil increases from τ = 2.8 h to 28 h, the optimal droplet diameter decreases from 275 to 100 µm (Fig. 2c). These variations in optimal droplet size notwithstanding, the very existence of an optimal droplet size is a fundamental consequence of accounting for the encounter rate.

### Monodispersed oil droplets

To examine the dynamics of oil droplet biodegradation in the natural environment, we developed MODEM (Supplementary information) to consider large numbers of droplets in parallel. This affects the time course and rate of overall oil biodegradation in two manners. First, the biodegradation of the overall oil mass proceeds through the biodegradation of many individual droplets, each initiated by an encounter stage whose duration is highly stochastic. Second, the growth and release of bacterial progeny from one droplet increases the encounter rate of other droplets, creating interactions among droplets. We account for both effects, considering a dispersion of oil droplets all having the same initial diameter (monodispersion).

**Figure 3:**
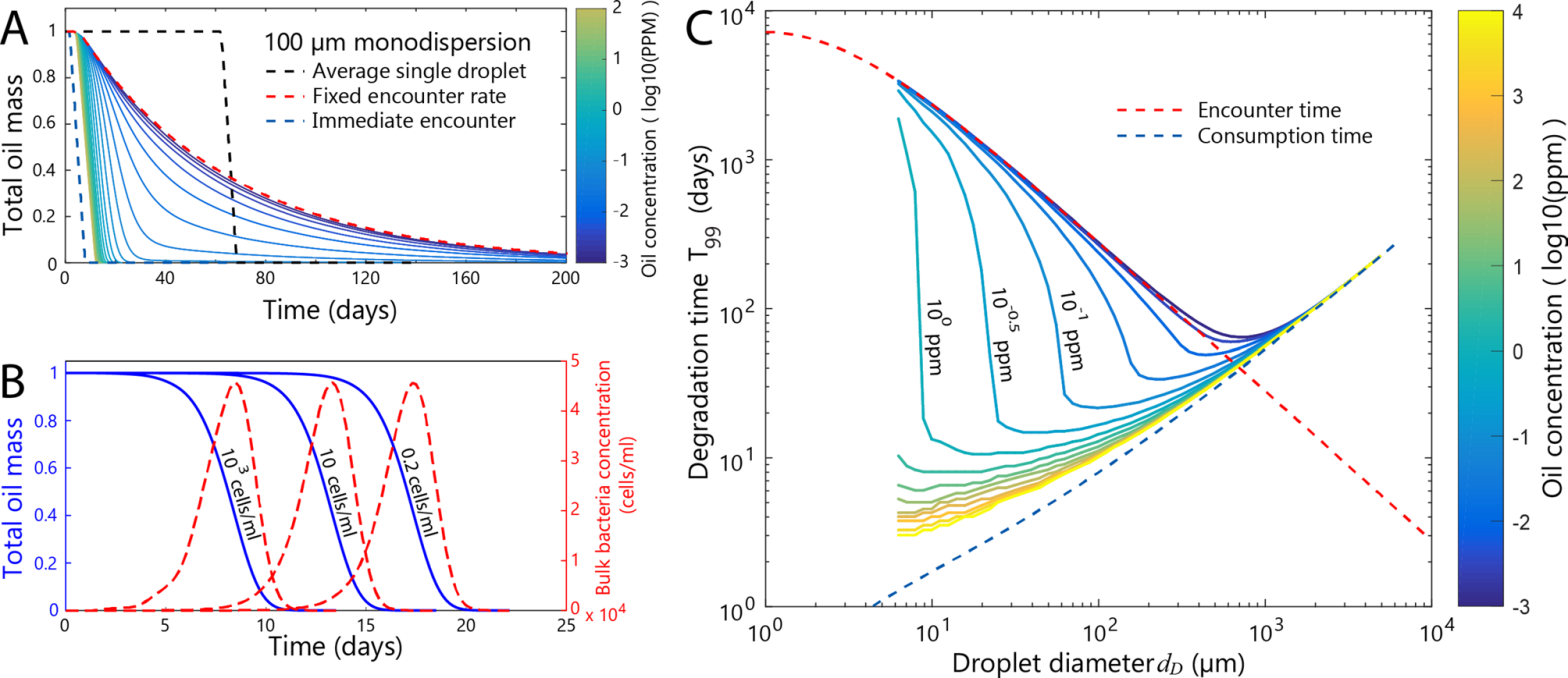
Degradation of monodispersed oil droplets. **(a)** The degradation of a single average droplet (black dashed line) overemphasizes the initial lag time and the suddenness of the degradation in comparison to the stochastic degradation of a dispersion of oil droplets. For a given droplet size (here, 100 µm diameter), the initial oil concentration (colormap) determines the extent of enrichment in the bulk bacterial concentration, which influences the overall degradation time. At very low oil concentrations (blue solid lines), the degradation of a monodispersion approaches an exponential decay limit of non-interacting droplets (red dashed line), for which the bulk bacterial concentration remains at its initial value. At high initial oil concentrations (yellow solid lines), the degradation is rapid and linear due to a large increase in bulk bacterial concentration, but delayed relative to the hypothetical case in which all droplets begin in an encountered state (blue dashed line). **(b)** The time course of the total oil mass for 20 µm diameter droplets at an initial oil concentration of 1 ppm (blue) and the bulk bacterial concentration (red) are given for three initial bacterial concentrations: 10^3^, 10, and 0.2 cells/ml. The initial background bacterial concentration alters the initial lag in the response, but the degradation curves and bacterial concentration profiles remain remarkably similar. **(c)** Optimal droplet size for the biodegradation of a monodispersion. The time *T*_99_ to degrade a monodispersion of droplets, as a function of the droplets’ diameter, for different initial oil concentrations (colormap). The encounter time (red dashed line) is the time for 99% of the oil droplets to be independently encountered. The consumption time (blue dashed line) is the time it takes for 99% of the mass of a droplet to be degraded after the first encounter occurs.

The biodegradation of a monodispersion of non-interacting (independent) oil droplets over time already differs dramatically from the biodegradation of an average droplet (Fig. 3a), because the encounter between bacteria and droplets is not a deterministic, but a random process. We model this encounter as a Poisson process based on independent bacteria among uniformly randomly distributed droplets. In a droplet dispersion, each individual droplet is randomly encountered and the overall biodegradation of oil requires the majority of the droplets to be encountered, making the dynamics of an average droplet a poor estimate of the total biodegradation. The biodegradation of a monodispersion of droplets shows a gradual onset, as some droplets are randomly encountered earlier than others, and a considerably longer tail compared to the average droplet, on account of some droplets being encountered only after a long time (Fig. 3a). For droplet sizes and bacterial concentrations where the encounter time is much larger than the consumption time (see Eq. 1), the biodegradation time is effectively governed by the time of first encounter of a droplet by a bacterium and one can effectively neglect the consumption time. In this regime, the total metabolizable oil mass decays exponentially (Fig. 3a, red dashed line) with a rate constant given by the encounter rate, λ.

The biodegradation of one droplet can affect other droplets through the common pool of planktonic bacteria (i.e., bacteria in the water column) and these interactions can result in an accelerated decrease in the total oil mass. When progeny bacteria are released into the water column from a droplet that has reached its saturation stage, the background bacterial concentration *C*_*B*_ in the water column increases and the expected encounter time of other, yet uncolonized droplets decreases (inversely with *C*_*B*_; Eq. 1). The resulting increase in encounter rate depends on the concentration of oil droplets, as each droplet can contribute a fixed number of bacteria to the environment. For a sufficiently high initial oil concentration, the first encountered droplets generate a strong positive feedback that leads to all droplets being rapidly encountered.

MODEM simulations of monodispersed oil droplets with varying initial oil concentrations (based on 100,000 droplets with 100 µm initial diameter, Fig. 3a) demonstrate a smooth transition between the non-interacting limit at low starting concentrations, and a nearly encounter-less biodegradation at very high starting concentrations. In order to capture the appropriate dynamics of the planktonic population, a constant rate of mortality (e.g., from predation; Supplementary information) was applied to bacteria not attached to droplets. For initial oil concentrations lower than 0.01 ppm, the time course of biodegradation is close to exponential and the time for 99% of the metabolizable oil to be degraded (*T*_99_ > 240 days) approaches the upper limit case of independent droplets (284 days). In stark contrast, for initial oil concentrations higher than 1 ppm the time course of biodegradation is close to linear, with an initial delay as the background bacterial concentration increases, and the 99% biodegradation time is *T*_99_ = 12–16 days. The initial delay is dependent on the starting concentration of bacteria in the water column (Fig. 3b). The considerable effect of oil concentration on biodegradation time highlights the importance of the droplet-bacteria encounter process.

For intermediate initial oil concentrations (0.01–1 ppm, Fig. 3a), the time course of biodegradation shows a very long tail with a distinct transition from the early biodegradation rate. Initially in this situation, the bacterial concentration in the water column increases due to the progeny shed by saturated droplets; this enhances encounters and causes the decay to be linear (with delay). However, this enhancement has a limited duration. As saturated droplets are degraded, fewer remain to sustain the higher bacterial concentration in the water column, which eventually returns to its baseline. As a result, the decay eventually becomes exponential where interactions among droplets are no longer significant. In this regime, the initial microbial degradation rate does not predict the final biodegradation time (*T*_99_).

Irrespective of the accelerated biodegradation due to droplet interactions, a clear optimal droplet size also exists for monodispersions (Fig. 3c), reflecting the same fundamental tradeoff between encounters and consumption found for the average droplet (Fig. 3c). The optimal droplet size is now dependent on the initial oil concentration (Fig. 3c). At low initial oil concentrations (< 0.01 ppm), droplets are degraded nearly independently and the optimal droplet diameter is approximately 700 µm. This is larger than the optimal size of the average droplet (275 µm), because we are now considering *T*_99_, requiring 99% of the droplets to be encountered by bacteria and degraded (Fig. 3c, red dashed line). For initial oil concentrations above 0.01 ppm, droplet interactions decrease the encounter time and shorten *T*_99._ Since it is harder for small droplets to cause the initial increase in encounter rate, even at high initial oil concentrations very small droplet dispersions degrade with independent encounters. However, for intermediate droplet sizes, the elevated starting oil concentration makes the overall biodegradation behave as if it is consumption-limited, greatly reducing *T*_99_ (Fig. 3c) and changing the optimal size_._ At an initial oil concentration of 0.01 ppm, the optimal diameter is approximately 400 µm, at 0.1 ppm it is 100 µm, and at 1 ppm it is 20 µm. This indicates that the tradeoff between encounters and consumption—and thus the optimal droplet size—depends on the oil concentration^18,22^. However, critically, for the low oil concentrations typically occurring in the field, the encounter-limited state applies and the smallest possible droplets are not the optimal droplets^39,42^.

### Polydispersed oil droplets

**Figure 4:**
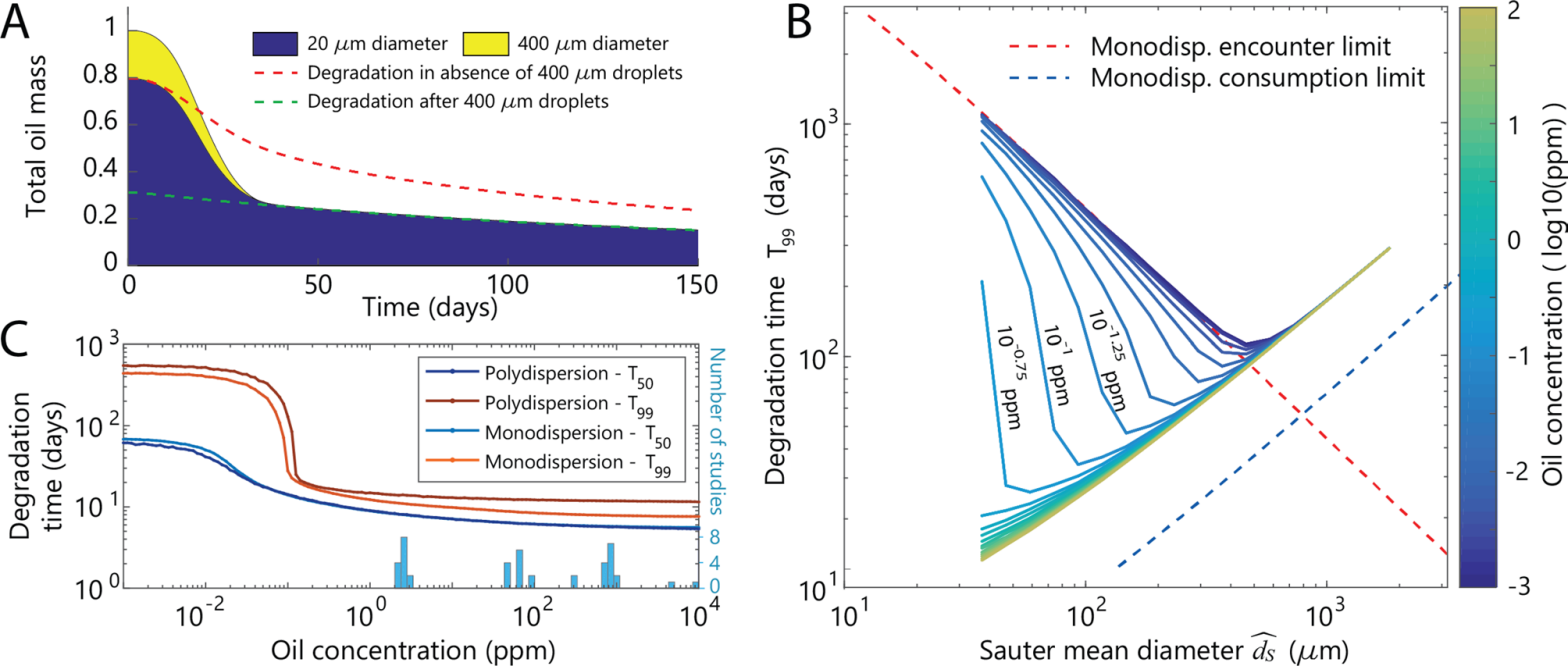
Degradation of polydispersed oil droplets. **(a)** In a polydispersion, feedback from larger droplets (through shedding of progeny bacteria) can accelerate the degradation of smaller droplets and thus the overall degradation. A two-size dispersion (solid shading) containing both 20 µm diameter (80% in mass) and 400 µm diameter (20% in mass) droplets degrades more rapidly than the 20 µm diameter droplets alone (red dashed line). After the 400 µm droplets are consumed, the dispersion continues to degrade at the same rate as a dispersion of only 20 µm droplets that had started with 31% of the initial oil concentration (green dashed line). **(b)** Polydispersions also exhibit an optimal droplet size, here expressed as an optimal Sauter mean diameter. For log-normal polydispersions (Supplementary Fig. 6) the dependence of the optimal Sauter mean diameter on initial oil concentration (colormap) is similar to that of monodispersions (Fig. 3c). The theoretical limit lines (dashed) are as in Fig. 3c. **(c)** For a given size (Sauter mean diameter 62 µm), the degradation time (*T*_99_) of a polydispersion is always longer than a monodispersion with the same diameter. The model considered 50,000 droplets. The transition to being encounter-limited (increased *T*_99_ at low starting oil concentration) also occurs at a slightly higher oil concentration for the polydispersion. The commonly used half-life metric (*T*_50_) shows very little difference between poly- and monodispersions, but also greatly underestimates the impact of encounter limitation. A histogram of the oil concentrations used in experimental studies recently reviewed^18^ is shown in light blue.

Due to waves, turbulence and jet breakup, droplet dispersions in natural environments have a broad range of sizes (10 µm to 10 mm in diameter)^39,41,43,44^, often modeled as a log-normal size distribution^41^. Larger droplets can reduce the overall biodegradation time in a polydispersion, even when comparatively sparse, due to their higher encounter rate with bacteria. To illustrate this cross-size interaction, consider a mixture of 400 µm and 20 µm diameter droplets, accounting for 20% and 80% of the oil mass, respectively. The 400 µm droplets are encountered and degraded first, releasing bacteria in the water column that accelerate the colonization of the 20 µm droplets (Fig. 4a). After the 400 µm droplets are consumed, the remaining 20 µm droplets are degraded exponentially at the encounter rate set by the background cell concentration. In this example, the addition of large droplets shortens *T*_99_ by 95 days and effectively reduces the mass of 20 µm droplets by 69% (Fig. 4a, green dashed line).

The encounter-consumption tradeoff also applies to natural polydispersions of oil droplets in the ocean, and optimal mean droplet sizes are comparable to those for monodispersions. We model natural droplet size distributions as a log-normal distribution in MODEM (Supplementary Fig. 6) characterized by the Sauter mean diameter ($\widehat{{d}_{S}}$), which is based on the average droplet surface-area-to-volume ratio^43^ (Supplementary information). In the consumption-limited regime (high $\widehat{{d}_{S}}$), the biodegradation time for a polydispersion is significantly larger than that of a monodispersion of droplets all having diameter $\widehat{{d}_{S}}$, due to the slow consumption of droplets larger than $\widehat{{d}_{S}}$ (Fig. 4b, black dashed line). In contrast, in the encounter-limited regime (low $\widehat{{d}_{S}}$), the biodegradation time is similar in the two cases (Fig. 4b, red dashed line). At an initial oil concentration of 0.01 ppm, the optimal Sauter mean diameter for minimizing biodegradation time remains approximately 400 µm, as for a monodispersion (but with *T*_99_ = 100 d), and at 0.1 ppm it is 100 µm (*T*_99_ = 35 d). The biodegradation time *T*_99_ for a polydispersion with small Sauter mean diameter ($\widehat{{d}_{S}}$< 200 µm) can be well approximated by modeling a monodispersion with diameter $\widehat{{d}_{S}}$ in MODEM (Fig. 4c).

## Discussion

Through a simple model incorporating microscale physics with the bacterial degradation of suspended insoluble oil droplets—MODEM—we have shown that the encounter rate of bacteria and oil droplets is a critical component of the biodegradation process and frequently an important determinant of the total biodegradation time in naturally relevant regimes. The tradeoff between the encounter time, which is smaller for larger droplets, and the consumption time, which is smaller for smaller droplets, leads to an optimal droplet size for which the oil biodegradation time is minimal. The existence of this optimal droplet size is a fundamental characteristic of the biodegradation process, independently of whether one considers the average droplet, a monodispersion of droplets or a polydispersion, and is in stark contrast to the paradigm that biodegradation rates simply increase with decreasing droplet size, based on surface-area-to-volume. The droplet-bacteria encounter process, previously overlooked in the analysis of oil biodegradation to the best of our knowledge, is therefore an important element in determining not only the time course of microbial degradation but also the optimal droplet size for degradation, when oil occurs in the form of droplets.

Our findings suggest that the biodegradation of oil droplets by bacteria in marine environments is in most instances limited by the encounter rate with oil droplets, not the consumption rate of droplets. This conclusion echoes recent calls to account for the rapid dilution that occurs in open waters^22^, which keeps oil concentrations low and inhibits increases in the concentration of oil-degrading bacteria in the water column to the same magnitudes observed in laboratory studies^45,46^. The limiting effect of encounters is potentially exacerbated by the higher rising rate of larger droplets, which reduces their prominence in a dispersion and limits their role as seeding grounds for the colonization of smaller droplets (Fig. 4a). For example, a plume of dispersed oil droplets and dissolved volatile hydrocarbons developed at 1100 m depth after the 2010 Deepwater Horizon incident^47^. Within the plume the concentration of oil reached 10 ppm^22^ and the concentration of bacteria was double (5.5 × 10^4^ cells/ml) that of the surrounding seawater^48^, possibly as a response primarily to dissolved components^49^. The majority of droplets inside the plume were less than 70 µm in diameter^22,39^. Outside the plume, oil concentrations were < 0.001 ppm^22^. Even if all bacteria in the plume had been oil-droplet degraders, it would have taken on average 10 days for 99% of a monodispersion of 50 µm diameter droplets to be encountered (43 days if droplets were 10 µm in diameter). At the same time, measurements of bacterial respiration in the Deepwater Horizon plume extending 35 km from the well site (equivalent to 5 days of transport by local currents) showed minimal signs of biodegradation^47^. Taken together with the results of our model, these observations support the conclusion that relatively few droplets were encountered by bacteria during their time in the suspended plume before dilution in the broader environment.

In the interest of focusing on the central underlying biophysical processes, we made several simplifying assumptions in the current implementation of MODEM. Oil droplets are assumed to be initially free of oil-degrading bacteria. This is likely a close approximation to the situation for dispersions created from the breakup of a jet at depth, where the large sudden increase in oil surface area and rapid translocation from a different environment make the likelihood of pre-attached oil-degrading bacteria from the water column very low when the droplets are first formed. While enhanced encounters with bacteria during the jet breakup or the coagulation of droplets may increase the effective encounter rates with oil-degrading bacteria, this would be limited to a brief time window when oil is initially introduced into the water column and is unlikely to affect the majority of droplets. A second assumption is that a single strain of bacteria is responsible for oil biodegradation, whereas in reality different strains are involved in hydrocarbon degradation^1^ and oil droplets are composed of multiple components that can be degraded at different rates^16^. This simplification is conservative, in that it likely underestimates the biodegradation time and exacerbates the encounter limitation: if biodegradation is partitioned across different bacteria, each strain will undergo a similar encounter constraint in parallel, extending the encounter process before all components can be biodegraded and increasing the optimal droplet size. For simplicity, the implemented model assumed that all droplets were neutrally buoyant. While this is a reasonable approximation for small droplets, larger droplets will rise to the surface in a size dependent manner. This can be partially taken into account in the current model implementation by shaping the droplet size distribution. Droplets rising would increase the encounter rate with suspended bacteria^50^ by a modest but appreciable factor, but would require spatially resolved distributions of bacteria in the water column, as larger droplets with higher rise rates would also release bacteria in to the water column well above the majority of the droplet dispersion.

There remain open questions on the details of how oil droplet-degrading bacteria interact with the oil–water interface over the full duration of the biodegradation process^51,52^. While many such bacteria are known to attach to, grow at, and deform the oil–water interface^26,27,29,30,32,53–57^, the model implementation described in this manuscript has by necessity made assumptions about the behavior of bacteria when the droplet surface saturates. The behavior of a hydrocarbon degrading bacteria detaching from the oil–water interface has been previously reported^28,35,37^. The mechanism involves the production of cell-bound biosurfactants that are used to disperse the oil phase, increase the interfacial area, and allow bacteria to detach and return to a planktonic state. However, some alternatives are explored in the Supplementary information. Different behaviors in this phase of a droplet’s biodegradation would primarily impact the consumption time (as in Fig. 2c). In the extreme, if attached bacteria were assumed to never leave the droplet, then the mechanism to increase the ambient concentration of bacteria would be broken, and the biodegradation time would be similar to the very low oil concentration curves in Figs. 3c and 4b.

The estimation of fluxes of hydrocarbons after a spill is difficult^3^ and current estimates may overplay the role of bacterial biodegradation of insoluble components in early stages. In marine environments it is challenging to distinguish between biodegradation and dilution and to differentiate the degradation of insoluble hydrocarbons from their dissolved counterparts. Often, the assertion of rapid biodegradation of droplets in the field has been based on comparisons to laboratory studies^18,20,34,46,48^. In light of the long biodegradation timescales predicted by our physically-based microscale model, alternative factors may be playing a larger role in the ultimate fate of suspended oil. For example, oil droplets may be locally removed by incorporation into marine snow^58,59^, bioaccumulation into higher organisms^60^, or by interactions with the sea floor. After the Deepwater Horizon spill, up to 31% of the oil plume was estimated to have settled into the nearby ocean sediments^61^. Once oil droplets are transported and diluted away from the plume, it is not clear whether encounters with marine snow or oil-degrading bacteria would be more frequent—this represents a further case of estimating and comparing encounter rates.

Biodegradation field measurements are challenging, but our modeling results reaffirm and provide a mechanistic basis for recent calls for caution in using laboratory bioremediation assays for guidance in determining mitigation strategies in the field^22^. MODEM results highlight the importance of the encounter process, which in turn depends strongly on oil concentration: the often high concentrations of oil used in enclosed experimental vessels in laboratory assays fail to capture the role of encounter dynamics in the natural environment, by skewing the biodegradation process to the often unnatural regime in which encounters are fast (Figs. 3c and 4c). Specifically, we determined that the starting oil concentrations in all of the 200 laboratory experiments summarized in a recent review^18^ are too high to observe encounter-driven delays in biodegradation (Fig. 4c, bar graph). We propose that this use of excessively high oil concentrations in laboratory experiments explains why the significance of the encounter stage between droplets and bacteria has been historically overlooked. In addition, the use of oil half-life as the parameter to characterize degradation in experiments^18,20^ misses or underrepresents the long tail in biodegradation (Fig. 3a). The discrepancy between the half-life (*T*_50_) and time for 99% degradation (*T*_99_) is particularly large at low starting oil concentrations (Fig. 4c). Our model results indicate that both factors will bias laboratory biodegradation studies towards overestimating oil degradation rates. The degradation of insoluble hydrocarbons by bacteria is an inherently microscale process that involves the physical interaction between bacteria and oil droplets. When studying oil bioremediation, adequately accounting for the physical environment should be considered with similar care as that devoted to the role of the chemical composition of the oil.

The importance of the encounter process in oil droplet biodegradation suggests a re-evaluation of some mitigation strategies aimed at increasing degradation rates during oil spills. A frequent argument for the use of chemical dispersants is the creation of more bio-available oil surface area by decreasing droplet size. The typical droplet size distribution measured after the addition of dispersants (diameter < 70 µm^20,42^) is well below the optimal size predicted by MODEM for most oil concentrations (Figs. 3c, 4b). As a consequence, the biodegradation of the small droplets created by the addition of dispersants will be limited by their encounter by bacteria. This can have a large effect on the overall biodegradation time, potentially increasing it by up to fifteen times (Figs. 3c, 4b). The results of MODEM indicate that mitigation strategies should aim to achieve a droplet size distribution that comes close to the optimal droplet size, as opposed to making droplets as small as possible. Large droplets in a polydispersion may accelerate the overall biodegradation (Fig. 4a), but this should be considered with caution due to turbulence- and buoyancy-driven dilution.

There are, of course, inherent challenges in achieving a specific, optimal, droplet size in the field, due ultimately to the limited control one has over droplet size, dependent on interfacial properties and fluid shear stresses, and the constraints posed by buoyancy. An alternative approach is to accelerate or bypass the encounter process. This could be achieved by seeding or otherwise promoting the growth of oil-degrading bacteria. This approach would be particularly effective near a spill site before significant dilution occurs^19^. In addition to having a smaller volume in which to augment the bacterial concentration, elevated local concentrations of oil would naturally increase the bacterial populations further as droplets in closer proximity act as incubators that favor the colonization of other droplets. Our results show that these droplet interactions can considerably ameliorate the encounter bottleneck. Propane and other labile oil components may naturally enhance the local oil-degrading bacterial populations^49^, but it is not necessarily the case that the same strains will degrade oil droplets. Artificial seeding (bio-augmentation) also has a long history with mixed success^62,63^. However, methods for locally increasing the number of oil-degrading bacteria prior to dilution take on renewed importance when encounters are a limiting component in bioremediation, as this work implies is the case for most ocean water column conditions.

In summary, we have shown that a mechanistic biophysical model can shed light on the fundamental microscale processes driving the biodegradation of oil droplets by marine bacteria and that these processes can have large impacts on the overall system, chiefly on the biodegradation time. While additional, often system-specific features will need to be taken into account, MODEM represents a fundamental basis for predicting the role of different processes in the biodegradation of oil in the sea, and may represent a useful tool in the complex task that is the management of an oil spill.

## Materials and methods

### Model implementation

The full description of the MODEM simulations capturing the encounter and biodegradation of individual droplets is available in the Supplementary information, and graphically summarized in Supplementary Fig. 1. All numerical simulations were done using Matlab (MathWorks Inc. version 2019a). The default model variable parameters are based on deep-sea conditions (Supplementary Table 1). The MODEM implementation described in this text assumes that there are smooth behavioral transitions (Supplementary Fig. 3) in the surface-attached growing bacteria as the surface approaches and exceeds saturation, which are detailed in the Supplementary information and analyzed against alternative forms.

## Supplementary Information

Supplementary Information.
