# Supplementary material for: A tradeoff between physical encounters and consumption determines an optimal droplet size for microbial degradation of dispersed oil
Source: Sci Rep. 2022 Mar 18;12:4734. doi: 10.1038/s41598-022-08581-7 (PMC8933409; doi:10.1038/s41598-022-08581-7)
Supplement: Supplementary file 1 — Supplementary Information. [file 41598_2022_8581_MOESM1_ESM.pdf]

Supplementary Information

A tradeoff between physical encounters and consumption determines an optimal droplet size for microbial degradation of dispersed oil

Authors

Vicente I. Fernandez[1], Roman Stocker[1*], and Gabriel Juarez[2*]

1. Bacterial and environmental parameters

Assuming that there are 10^6 droplets per liter in an environment, the distance between droplets can be roughly approximated by considering a lattice of evenly spaced droplets forming a cube with volume 10^6 mm^3 (1 liter). In this uniform distribution, the spacing between droplets would be 1 mm. If droplets are considered to be randomly distributed within the volume, the average distance from one droplet to the next closest droplet will be smaller than 1 mm [1].

Calculations for the average time it takes a bacterium to encounter a droplet are determined by a diffusive encounter model based on the number of droplets in a given volume, as opposed to the distance between droplets. The formula for the average time to encounter a droplet is analogous to the encounter component of equation 1 of the main text:

$$T_{avg,bacteria} = \frac{1}{2\pi(d_B + d_D)(D_B + D_D)C_D}$$

Where C_D is concentration of oil droplets in the environment (#/volume), and the other variables are as in the main text. This model for encounter rates has a history of use in the environmental microbiology field [2]. Bacteria are modeled as diffusive objects with an effective diffusivity that incorporates any random-walk type of motility. Alternative approaches can further account for additional advective flux [3] (such as with rising/sinking droplets) or for swimming-flow interactions [4], but for this manuscript we have restricted our analysis to the fundamental diffusive encounter model that minimizes the need for other assumptions. For the implementation of MODEM, we have assumed that bacteria have an effective spherical diameter of 1 µm, based on a rod-shaped morphology (cylindrical body with spherical endcaps) with a 2 µm length and 0.6 µm diameter. The diffusivity for non-motile bacteria and oil droplets is then calculated using the Stokes-Einstein equation:

$$D = \frac{k_B T}{3\pi\eta d},$$

where k_B is Boltzmann's constant, T is the temperature, η is the viscosity of water, and d is the object diameter. In the case of motile bacteria, the bacterial diffusivity D_B is inferred from their long-term behavior [5]. For the calculation in the manuscript introduction, we use an effective diffusivity of 10^{-10} m^2/s, based on observations of motile marine bacteria [6].

Variations in marine bacteria size and shape will affect certain components of the modeled biodegradation process along the axes shown in Fig 2B&C, possibly making small shifts to the predicted optimal droplet size for minimizing biodegradation time.

Oil degrading bacteria have been found to generally correspond in volume to that of other surface-attaching copiotrophic marine bacteria (0.91 – 1.39 µm Equivalent Spherical Diameter) [7]. *Alcanivorax borkurnensis* was found to range from 1.09 to 1.55 µm [8], *Alcanivorax mobilis* from 0.38 to 0.89 µm [9], and *Oleiphilus messinensis* from 0.77 to 1.07 µm depending on whether it was attached to oil [10].

As a representative size, the model implementation shown here uses a 1 µm ESD (Supplementary Table 1). The size influences both the diffusivity of planktonic bacteria (affecting encounter rates with oil droplets) and the maximum number of cells that can attach to a droplet surface. The former would have impacts analogous to that shown in Fig. 2B, with smaller cells leading to faster encounters. For a fixed doubling time (growth rate), the maximum surface population has smaller impact on a droplet's biodegradation since the metabolized carbon is proportional to cell biomass increase, not population.

Bacteria are most commonly spherical or rod shaped, with rod shaped bacteria having a typical aspect ratio of approximately 3. *A. mobilis*, *A borkurnensis*, and *O. messinensis* were all described to have an aspect ratio of less than 3 [8]–[10]. For these low aspect ratios, the impact of shape on diffusivity, and therefor droplet encounter rates, is small [11], and the diffusivity is reasonably approximated by a sphere with the same volume.

2. Experimental details for images used as motivation for MODEM in Figure 1

Inset images are a projection of the mid-plane of a crude oil droplet that is 100 µm in diameter after exposure to oil degrading bacteria *Alcanivorax borkumensis*. The crude oil used was a light crude oil from the Macondo Prospect, MC252. Using custom microfluidic chambers, the crude oil droplet can be held stationary over the duration of 48 hours. Time-lapse phase contrast microscopy was used to directly visualize cell encounters, cell growth, and colonization of the droplet.

3. MODEM implementation

The Microscale Oil Degradation Model (MODEM) simulates the degradation of suspended oil droplets by attached bacteria, factoring in the size distribution of droplets, the dynamic concentration of bacteria in the environment, and the random encounters between bacteria and

droplets. At the heart of the MODEM is a Monte-Carlo style simulation in which a large number (10,000 to 1,000,000) of individual droplets are tracked over time. These droplets are taken from a discretized size distribution (see Supplementary Figure 6) with diameters ranging from 10 µm to 400 µm with increments of 2 µm. The timestep of the simulation is one hour.

The implementation of MODEM is accomplished in two steps in order to minimize the computation time. First the consumption process is pre-calculated for all droplet sizes starting from the point of the first oil-degrading bacterium attaching to the droplet surface. The degradation after encounter is modeled as following a deterministic trajectory. In the second step, the stochastic encounters are simulated and tracked, referring back to the consumption dynamics to update the bulk bacterial concentration and the total remaining oil.

The simulation of the degradation of a droplet dispersion was implemented in Matlab, and begins by creating a vector of droplet sizes, with each element representing a single droplet's size. A matching vector records the time for the first bacterial encounter of each droplet, which initially has all of the droplets un-encountered. The simulation follows in a loop in time (Supplementary Figure 1), until time runs out or the remaining metabolizable oil is below a threshold (e.g. <0.1% of starting metabolizable oil volume). Each step of the loop proceeds as follows:

A. Identify un-encountered droplets.
B. Update the encounter rate for each droplet size based on the current bulk concentration of bacteria.
C. Generate uniformly distributed random numbers for each un-encountered droplet and compare to a threshold based on the probability of an encounter occurring in the Δt timestep
D. For every successful encounter, the current time is entered into the vector of encounter times.
E. For every previously encountered droplet, the remaining metabolizable oil and the number of new planktonic bacteria released in the timestep are calculated based on the pre-computed consumption dynamics and the time between the current time and the encounter time for that droplet. From these, the total remaining metabolizable oil and the total addition to the free-swimming bacteria is computed for the timestep.
F. Update the bulk concentration of bacteria for the next timestep based on turnover and contributions from degrading oil droplets.

In the following sections, we will elaborate on the precomputed consumption dynamics and the stochastic encounters.

4. Degradation dynamics

Once the first oil-degrading bacteria encounters and attaches to an oil droplet, the bacteria will convert the metabolizable oil into new biomass. The total amount of metabolizable oil in a

droplet prior to degradation by bacteria is denoted M_o, and is expressed in units of number of bacteria, corresponding to how many bacteria are produced through the consumption of this oil. This conversion is calculated via the carbon content of oil droplets and bacteria (Supplementary Table I), including the biological growth efficiency which accounts for carbon lost in respiration.

For the MODEM, the variables of interest are both the amount of metabolizable oil remaining in an oil droplet at a given time after colonization and the rate of new cells being released into the environment. In order to evaluate these terms, the changing size of the oil droplet and the number of bacteria on the droplet surface are tracked. These variables are simulated until the metabolizable oil is entirely degraded, in increments of 1 minute (Supplementary Figure 2).

The growth of bacteria on the oil droplet surface is exponential, with the change in the number of bacteria given by:

$$\Delta N = \lambda N f \Delta t$$

Where N is the number of attached bacteria, λ is the bacterial growth rate, Δt is the time step, and f is the fraction of new cells that remain attached. Early in the consumption process, the growth of attached bacteria is unbounded and $f = 1$. However, when a droplet becomes fully colonized, all new bacteria disperse into the environment and $f = 0$. The transition to a fully colonized droplet is made smoothly as a function of the surface area coverage fraction, which is the fraction of the instantaneous surface area (changing as the droplet shrinks) that is covered by the attached bacteria. For the MODEM presented in this manuscript, the transition was chosen such that all new bacteria disperse when the bacteria comprise 1.5 × the surface area (Supplementary Figure 3). A surface area coverage fraction greater than 1 was chosen since bacteria can develop biofilms. The specific form of the fraction of new cells attached is:

$$f = 0.5\,(1 - \tanh(5\,SR - 5.25))$$

Where SR is the surface coverage fraction. The function is plotted in Supplementary Figure 3 (blue). Note that the surface area of the droplet is calculated using the entire volume of the oil droplet, not just the metabolizable oil component.

As a result of this formulation, the number of bacteria added to the environment from a single droplet (per Δt) is $G = \lambda N(1 - f)$. This rate of bacterial dispersal provides the feedback that allows one droplet to influence the degradation of another, but altering the time necessary for its encounter. In order to minimize numerical artifacts in the simulation, the rate of bacterial dispersal is smoothly returned to zero as the metabolizable oil reaches zero (see Supplementary Figure 2, yellow).

According to this model, the number of attached bacteria stabilizes at fixed maximum value B_{max} (Supplementary Figure 2, purple) and the consumption of metabolizable oil proceeds linearly after an initial growth phase (Supplementary Figure 2, blue). This continues until the remaining metabolizable reaches zero, at which point the consumption and growth stop. For the

analytical estimate of the average degradation time in the main text, a simpler B_{max} based on 1.5× the initial droplet surface area was used instead of the simulated results.

5. Alternative consumption dynamics

It is worth briefly considering an alternative to the degradation dynamics we have chosen for the MODEM, in which the maximum number of bacteria attached to the droplet surface is proportional to the surface area as it evolves over time. In the standard MODEM dynamics, the number of bacteria attached to the surface of a droplet continue until they reach a maximum value, after which all new bacteria disperse but any bacteria that were attached remain attached. This results in a surface area coverage fraction that increases even after no new bacteria are being added to the surface. This was chosen to model biofilm formation on droplets. However, if bacteria do not form biofilms, then one would expect that the number of attached bacteria would shrink as the size of the droplet shrinks, since the reduction in surface area would push out some of the previously attached bacteria.

We investigated this alternative model by altering the function for the fraction of new cells that remain attached, allowing it to express negative values. Specifically, the previous function for f was simply extended linearly from its inflection point (Supplementary Figure 3, red). As expected, this results in different consumption dynamics. The number of attached bacteria decreases after initially saturating the droplet surface (Supplementary Figure 4, purple), leading to oil consumption that slows over time. As a consequence of the changing consumption rate, and the fact that the peak number of bacteria attached is lower than the standard model, the overall degradation time is increased by the alternative surface coverage model (compare Supplementary Figures 2 and 4).

Despite the increased time for consumption generated by the alternative surface coverage model, it does not meaningfully effect the interaction between encounters and consumption of oil droplets. Supplementary Figure 5 depicts the two times for degradation, based on the standard and alternative consumption models. In both cases, the essential tradeoff remains, with the crossover point being reduced by less than a factor of two in droplet diameter with the slower consumption model.

6. Stochastic encounters

Once the droplet consumption dynamics are specified for all potential droplet sizes, the full stochastic degradation model generates and tracks the first encounter of each droplet in the simulation. Importantly, the rate of encounters varies both depending on the droplet size and over time. As a result, the stochastic portion of MODEM steps through time, updating the arrival rates and simulating encounters as it progresses. The timestep for this stage was one hour. For the

consumption dynamics, a shorter timestep was used to accurately capture the initial rapid bacterial growth and the endpoint growth cessation. In modeling the droplet encounters the constraint is that the probability of two encounters occurring within a timestep for any droplet size is minimal.

In line with equation 1 of the main text, the rate of encounters that a droplet of size d_D experiences is:

$$\lambda = 2\pi(d_B + d_D)(D_B + D_D)C_B \,.$$

In the model simulation, each droplet has a defined diameter that is static prior to the first encounter with oil-degrading bacteria. C_B is the bulk concentration of bacteria, discussed further below, which changes over time due to the degradation of oil droplets. Following the assumption that bacteria are randomly distributed in the fluid and acting independently, the process of bacteria encountering an oil droplet can be approximated as a Poisson arrival process. This implies that the time until the next arrival of a bacterium is exponentially distributed with rate λ. Importantly for the simulation, it also implies that for small enough increments in time, the probability of an encounter occurring is independent of any other timestep and approximately equal to $1 - e^{-\lambda\Delta t}$.

The concentration of oil becomes relevant when it is necessary to update the concentration of free-swimming bacteria in the bulk fluid. Since the number of droplets, and therefore the oil volume, is limited by computational ability, it remains fixed. Instead different oil concentrations imply different control volumes for the simulation of the oil droplets. When new bacteria are released from oil droplets in the later stages of their degradation, they are assumed to disperse into that same volume. In this way, large control volumes reduce the effect that oil droplet degradation has on the bulk bacterial concentration. The following equation is used to update the number of bacteria in the control volume from one timestep to the next:

$$N_{k+1} = \left(\frac{\tau - \Delta t}{\tau}\right) N_k + \frac{\Delta t}{\tau} N_0 + \Delta N_{droplets} \,,$$

where N_k is the number of bacteria in the control volume at timestep k, N_0 is the baseline number of bacteria in the control volume, and $\Delta N_{droplets}$ is the total new bacteria added to the control volume from oil degradation. The parameter τ is the turnover time (24 hours for the simulations in this manuscript), which generates an exponential decay back to the bacterial concentration baseline in the absence of inputs from oil degradation. The update equation above is a discretized version of $\frac{dN}{dt} = -\frac{N}{\tau} + J_{droplets}$, where $J_{droplets}$ is the rate of new bacteria entering the control volume from droplets.

7. Droplet polydispersions

Droplet polydispersions can be characterized by a number ensemble metrics including the mean volume, mean surface area, and mean radius [12]. The mean quantity characterizing a

distribution is often expressed as a droplet diameter that corresponds to the mean quantity (e.g. diameter of a droplet with the mean volume). We characterize a polydispersed droplet distribution by the Sauter mean diameter, defined as the diameter of a droplet that has the same surface-area-to-volume-ratio as the entire polydispersion [12], given by

$$\widehat{D_S} = \frac{6 \int {}^{4}\!/_{3}\, \pi r^3 P(r) dr}{\int 4\pi r^2 P(r) dr},$$

where r is the droplet radius and $P(r)$ is the droplet size distribution. The Sauter mean of a monodispersion is the same droplet diameter, and for lognormal distributions, a larger mean diameter also implies a larger Sauter mean diameter. The Sauter mean diameter was chosen due to the importance of the surface-area-to-volume ratio in the consumption stage of droplet degradation, and because it was found to have a reasonable correspondence in degradation times between monodispersion and polydispersion degradation (see Fig. 4(c)).

To examine the degradation of polydispersions with MODEM, we used a family of lognormal droplet size distributions depicted in Supplementary Figure 6 and characterized by a constant standard deviation of the logarithmic droplet radii (0.5) and a varying mean of the logarithmic droplet radii. The mean values in Supplementary Figure 6 and Fig. 4(b) are specified by log(5:5:80). The Sauter mean droplet diameters were calculated for the discrete implementations of the droplet polydispersions. For this family of size distributions, the Sauter mean diameter is directly proportional to the mean diameter, the diameter with mean surface area, and the diameter with mean volume.

References

[1] R. Stocker, "The 100 μm length scale in the microbial ocean," *Aquat. Microb. Ecol.*, vol. 76, no. 3, pp. 189–194, 2015.

[2] T. Kiørboe, *A Mechanistic Approach to Plankton Ecology*. Princeton, New Jersey: Princeton University Press, 2008.

[3] L. Karp-Boss, E. Boss, and P. Jumars, "Nutrient fluxes to planktonic osmotrophs in the presence of fluid motion," *Oceanogr. Mar. Biol.*, vol. 34, no. May 2014, pp. 71–107, 1996.

[4] J. Słomka, U. Alcolombri, E. Secchi, R. Stocker, and V. I. Fernandez, "Encounter rates between bacteria and small sinking particles," 2019.

[5] T. Ahmed and R. Stocker, "Experimental verification of the behavioral foundation of bacterial transport parameters using microfluidics.," *Biophys. J.*, vol. 95, no. 9, pp. 4481–93, Nov. 2008.

[6] T. Kiørboe, H. P. Grossart, H. Ploug, and K. Tang, "Mechanisms and rates of colonisation of sinking aggregates," *Appl. Environ. Microbiol.*, vol. 68, no. 8, pp. 3996–4006, 2002.

[7] S. Kjelleberg, K. C. Marshall, and M. Hermansson, "Oligotrophic and copiotrophic marine bacteria-observations related to attachment," *FEMS Microbiol. Lett.*, vol. 31, no. 2, pp. 89–96, 1985.

[8] M. M. Yakimov *et al.*, "Alcanivorax borkumensis gen. nov., sp. nov., a new, hydrocarbon-degrading and surfactant-producing marine bacterium," *Int. J. Syst. Bacteriol.*, vol. 48, no. 2, pp. 339–348, Apr. 1998.

[9] S. Yang, M. Li, Q. Lai, G. Li, and Z. Shao, "Alcanivorax mobilis sp. Nov., a new hydrocarbon-degrading bacterium isolated from deep-sea sediment," *Int. J. Syst. Evol. Microbiol.*, vol. 68, no. 5, pp. 1639–1643, 2018.

[10] P. N. Golyshin, Ta. N. Chernikova, W. R. Abraham, H. Lünsdorf, K. N. Timmis, and M. M. Yakimov, "Oleiphilaceae fam. nov., to include Oleiphilus messinensis gen. nov., sp. nov., a novel marine bacterium that obligately utilizes hydrocarbons," *Int. J. Syst. Evol. Microbiol.*, vol. 52, no. 3, pp. 901–911, 2002.

[11] D. B. Dusenbery, "Fitness landscapes for effects of shape on chemotaxis and other behaviors of bacteria," *J. Bacteriol.*, vol. 180, no. 22, pp. 5978–5983, 1998.

[12] F. Chen and P. D. Yapa, "Estimating the Oil Droplet Size Distributions in Deepwater Oil Spills," *J. Hydraul. Eng.*, vol. 133, no. 2, pp. 197–207, 2007.

[13] R. C. Prince, J. D. Butler, and A. D. Redman, "The Rate of Crude Oil Biodegradation in the Sea," *Environ. Sci. Technol.*, vol. 51, no. 3, pp. 1278–1284, 2017.

[14] T. C. Hazen *et al.*, "Deep-sea oil plume enriches indigenous oil-degrading bacteria.," *Science*, vol. 330, no. 6001, pp. 204–8, Oct. 2010.

[15] M. Loferer-Krößbacher, J. Klima, and R. Psenner, "Determination of bacterial cell dry mass by transmission electron microscopy and densitometric image analysis," *Appl. Environ. Microbiol.*, vol. 64, no. 2, pp. 688–694, 1998.

[16] K. Vrede, M. Heldal, S. Norland, and G. Bratbak, "Elemental Composition (C , N , P) and Cell Volume of Exponentially Growing and Nutrient-Limited Bacterioplankton," *Appl.*, vol. 68, no. 6, pp. 2965–2971, 2002.

[17] P. A. del Giorgio and J. J. Cole, "Bacterial Growth Efficiency in Natural Aquatic Systems," *Annu. Rev. Ecol. Syst.*, vol. 29, no. 1, pp. 503–541, 1998.

[18] P. S. Daling, P. J. Brandvik, D. Mackay, and O. Johansen, "Characterization of crude oils

for environmental purposes," *Oil Chem. Pollut.*, vol. 7, no. 3, pp. 199–224, 1990.
[19] C. M. Reddy *et al.*, "Composition and fate of gas and oil released to the water column during the Deepwater Horizon oil spill," *Proc. Natl. Acad. Sci.*, vol. 109, no. 50, pp. 20229–20234, 2012.
[20] P. S. Stewart, D. J. Tedaldi, A. R. Lewis, and E. Goldman, "Biodegradation rates in seawater of crude oil," *Water Environ. Res.*, vol. 65, no. 7, pp. 845–848, 1993.

Figures

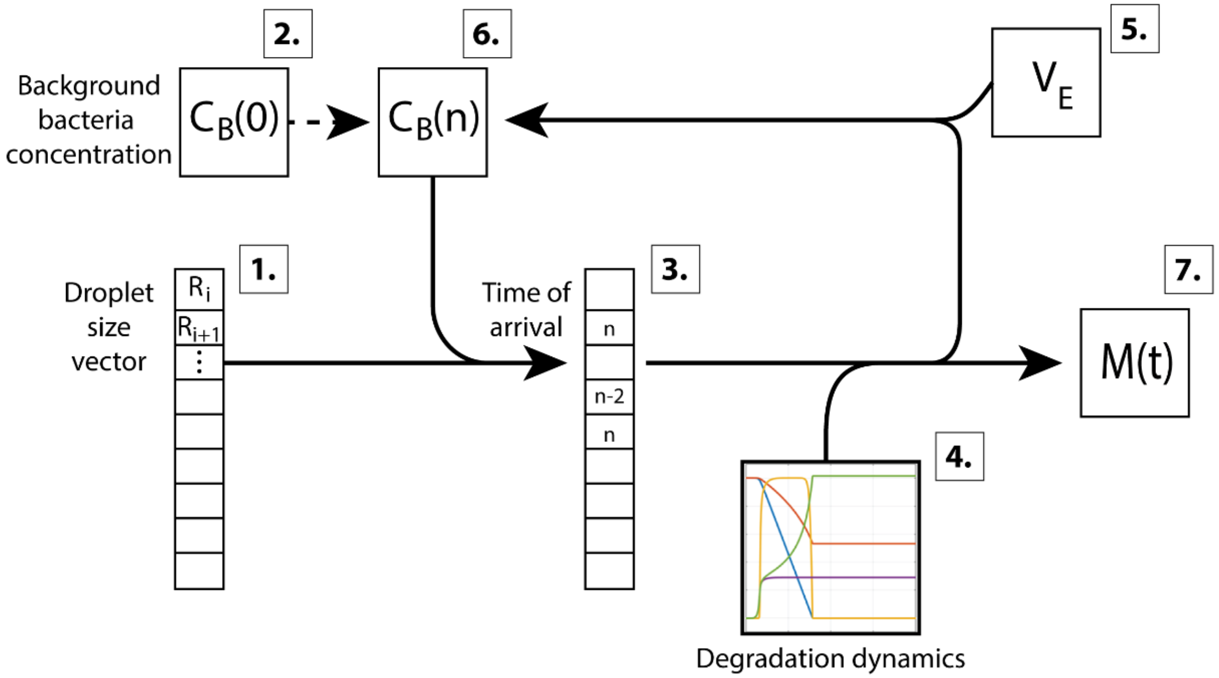

Supplementary Figure 1. MODEM simulation diagram. At the start of the simulation (1), a predefined number of droplets are assigned sizes randomly drawn from a preset distribution and recorded in a vector. Here, i indicates the droplet index. The background environment concentration of bacteria is also specified (2), and in conjunction with the droplet size vector, a random number is generated for each droplet to determine if it encountered by a bacterium in the first timestep. The probability of encounter depends on the size of the individual droplets. Another vector (3) records the integer timestep in which the first encounter occurs. The time difference between the current timestep and the time of encounter for each particle is used in combination with predefined consumption dynamics (4) to identify how many new bacteria are released into the environment during the current timestep. Taken together with the modeled volume (V_E, determined by the starting oil concentration) the number of newly dispersed bacteria is used to update the background bacteria concentration (6). Quantities such as the total remaining oil volume (7) can then be characterized over time directly from the vector of encounter times with the degradation dynamics.

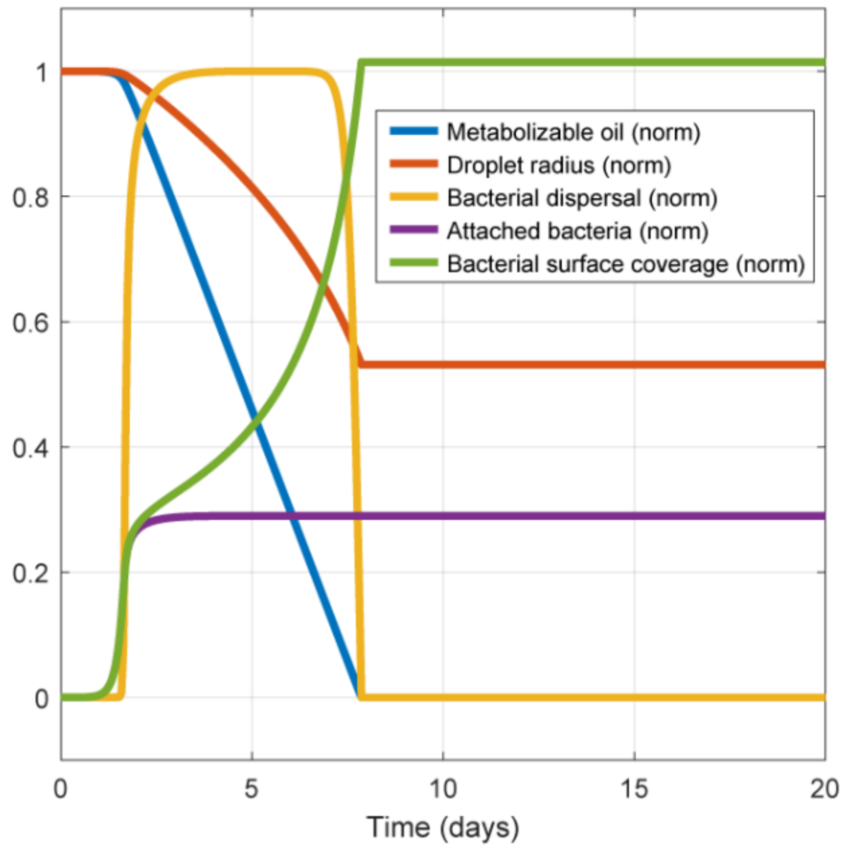

Supplementary Figure 2. Standard MODEM degradation dynamics. Once the first oil-degrading bacterium attaches to an oil droplet, the growth, degradation, and dispersal dynmaics proceed deterministically for each droplet size. In this standard model, the attached bacteria (purple) reach and maintain a maximum number, which results in linear oil degradation (blue).

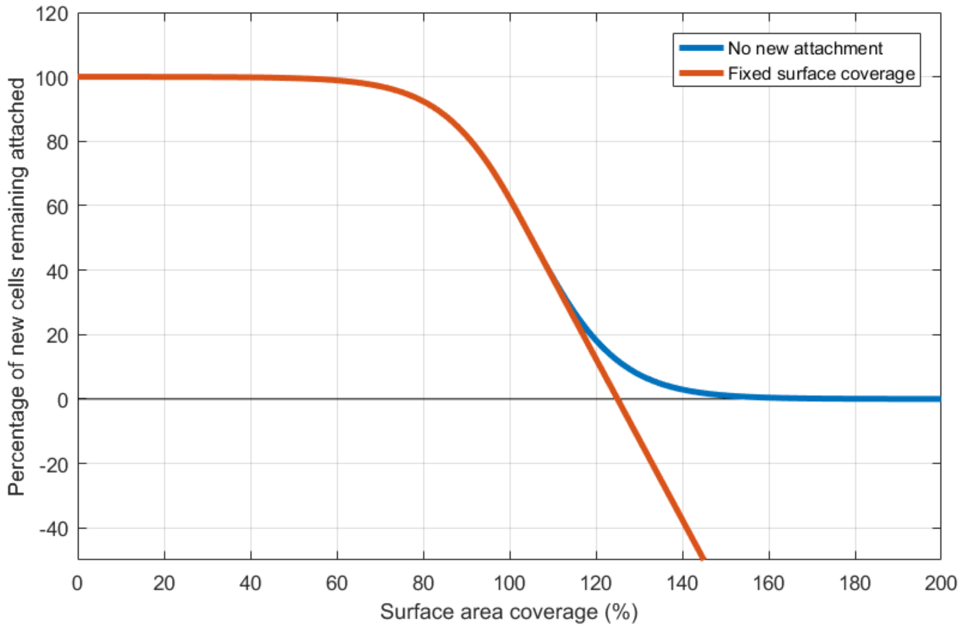

Supplementary Figure 3. Daughter cell dispersion. Each timestep in MODEM, a percentage of the new cells that grow from the degradation of an oil droplet remain attached to the droplet. The remaining are released into the environment with the possibility of encountering and degrading other droplets. While the population of bacteria on a droplet is small, MODEM assumes that nearly all progeny remain attached. As the attached bacteria grow and cover a significant portion of the droplet surface area, the percentage of new cells that remain attached smoothly transitions to zero in the standard implementation (blue line). This leads to a fixed number of bacteria remaining attached. In an alternative implementation (red line), parent cells will also leave the droplet to maintain a fixed surface coverage.

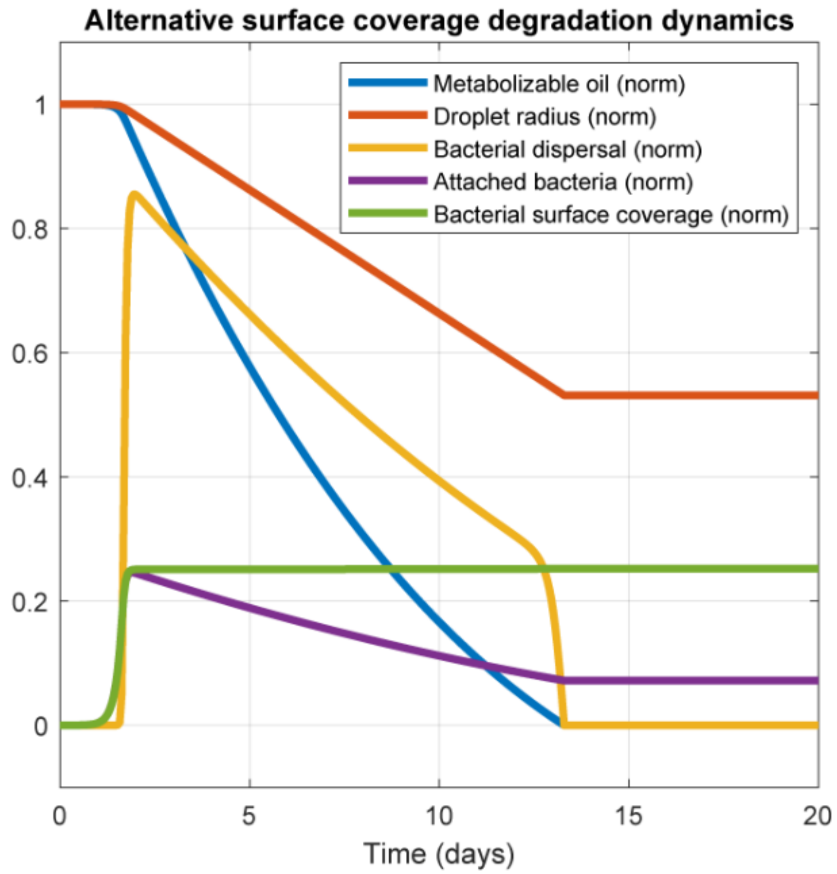

Supplementary Figure 4. Dynamic surface coverage degradation dynamics. This plot depicts elements of the deterministic degradation dynamics that occur after the initial colonization of a droplet by an oil degrading bacterium. In this alternative scenario, a maximum surface coverage (green) is enforced as the droplet shrinks during degradation. As a result, the number of attached bacteria (purple) shrinks after an initial saturation, and the oil degradation (blue) slows as the droplet size shrinks.

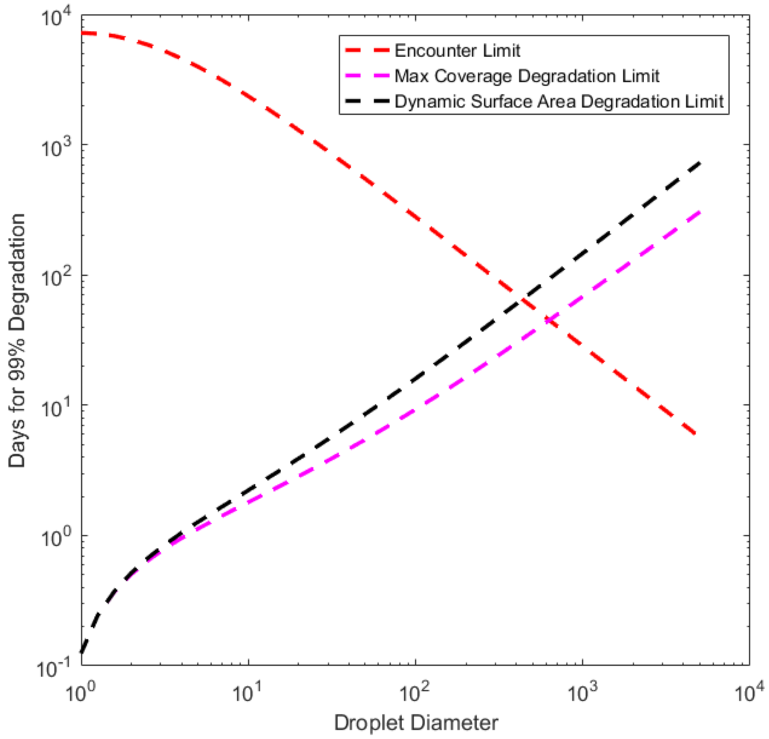

Supplementary Figure 5. Comparison of surface coverage impact on degradation. In the context of a droplet monodispersion (compare with Fig. 3(c)), both models of the activity of bacteria at the droplet interface when it reaches capacity result in a tradeoff between encounters and consumption, with a similar transition droplet size. The maximum coverage model is the one implemented in the manuscript text, where bacteria grow on the surface until capacity is reached and then maintain that number during the droplet degradation. The dynamic surface area model assumes a maximum surface area concentration for bacteria, resulting in a slowing consumption as the droplet diameter shrinks.

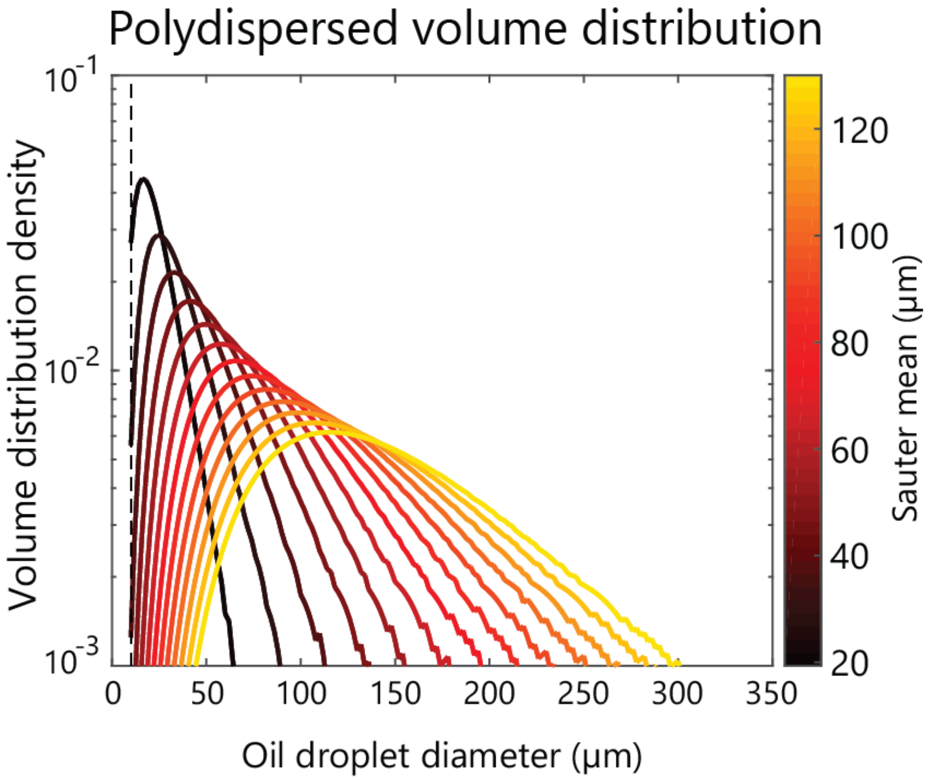

Supplementary Figure 6. The volume distribution for simulated log-normal droplet polydispersions. The line coloring indicates the Sauter mean diameter for each distribution. These droplet distributions are the basis of the simulations in Fig. 4(b).

Supplementary table

Supplementary Table I. **Model parameters.** In the absence of specific statements describing variations (e.g. Figure 2), the following parameters were used for the MODEM implementation. The equivalent bacterial diameter is calculated based on the equivalent spherical diameter of a rod-shaped bacterium 0.6 µm thick and 2.0 µm long. Values we have chosen are within the range of experimental measurements with corresponding references.

Symbol	Description	Value	Units	References
d_B	Equivalent bacterial diameter	1.0	µm	
d_D	Droplet diameter	1 - 300	µm	
C_o	Oil concentration	10^{-3} - 10^{4}	ppm	[13]
C_B	Bulk concentration of oil-degrading bacteria	10^{3}	cells/ml	[14]
τ	Bacteria doubling time	10^{4}	s	
m_B	Carbon content per cell	100×10^{-15}	g_C	[15], [16]
Γ	Bacterial growth efficiency	0.5		[17]
T	Temperature of seawater	283.15	K	
η	Dynamic viscosity of seawater	1.03×10^{-3}	Pa s	
ρ	Oil density	850	kg/m^3	[18], [19]
	Metabolizable carbon in oil	85	%	[19], [20]
